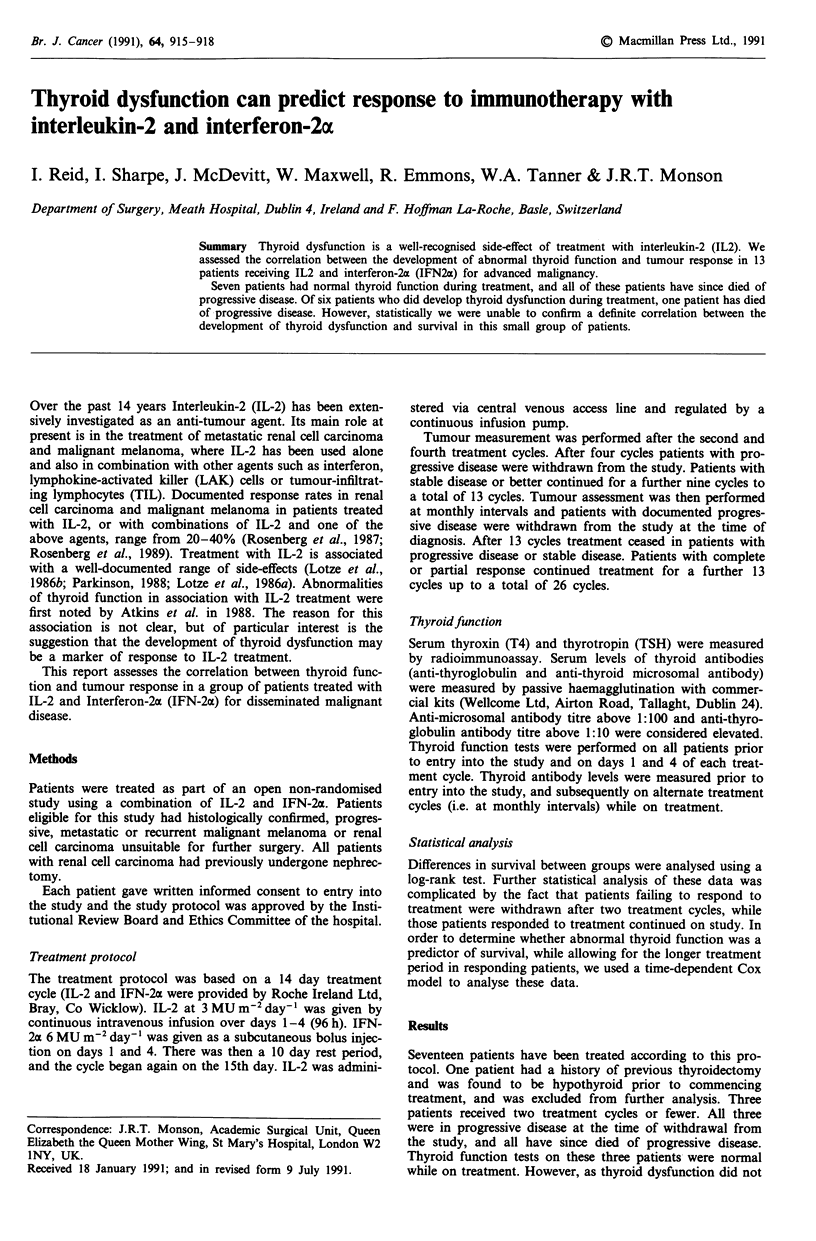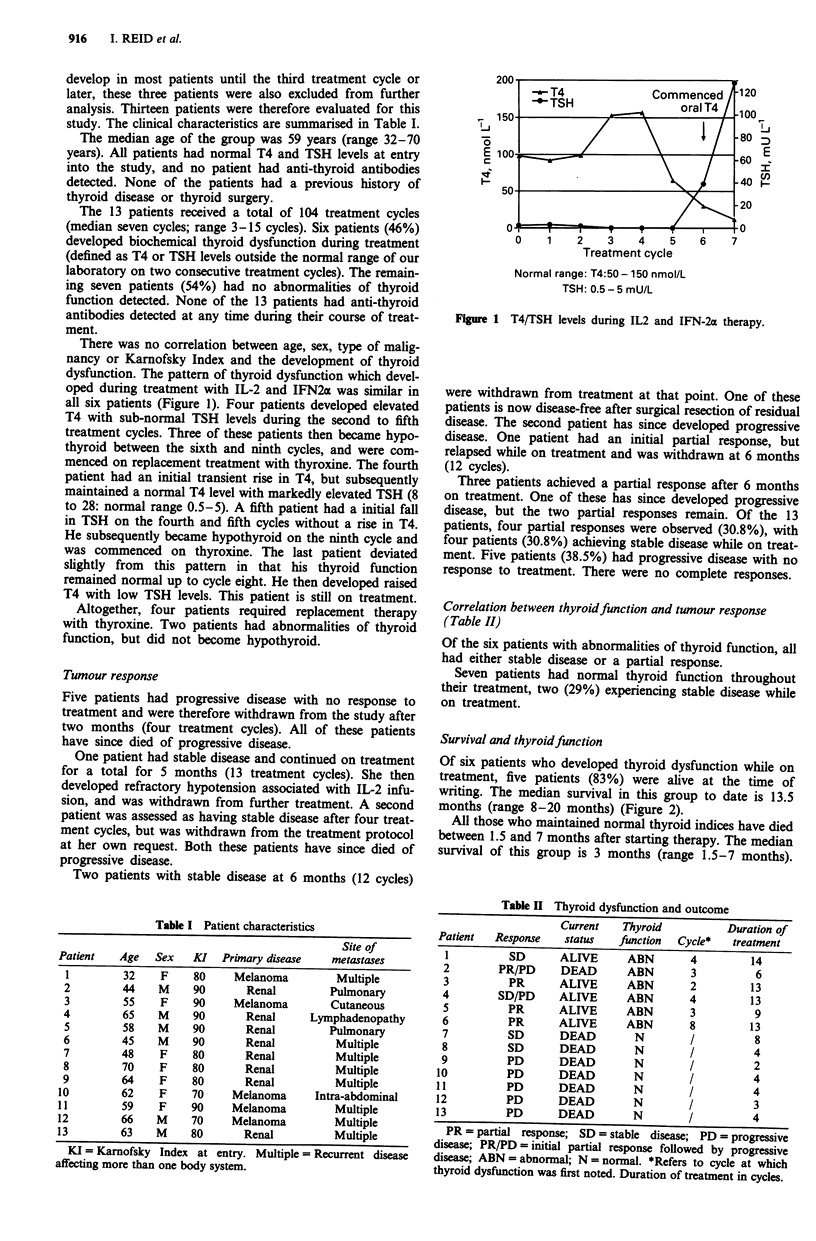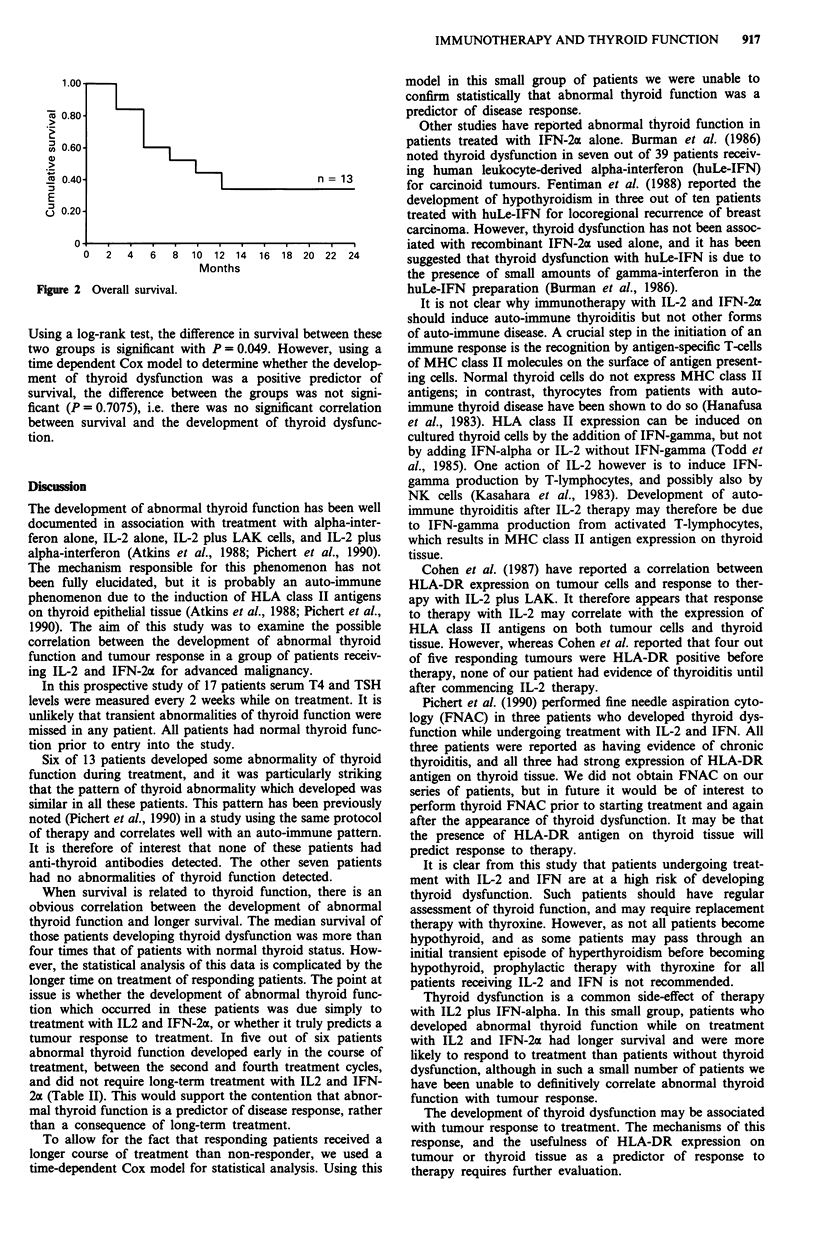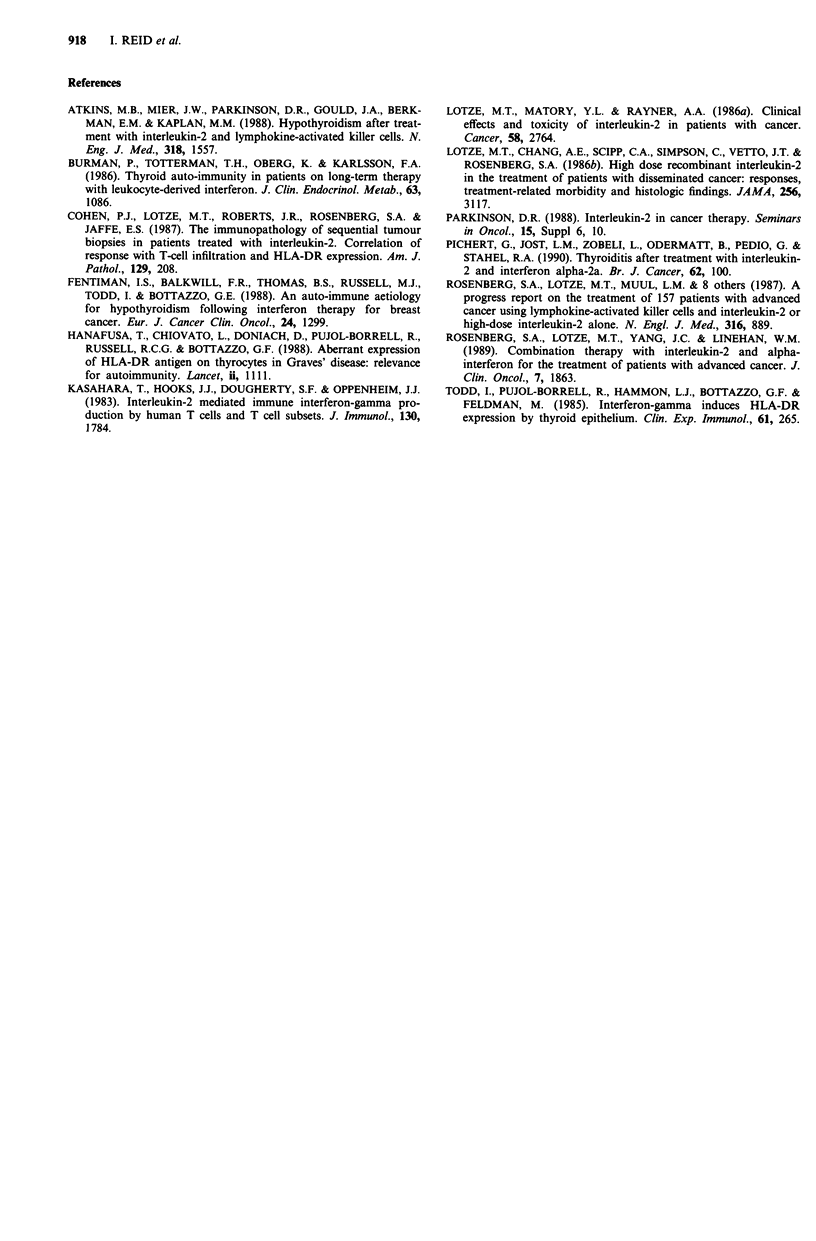# Thyroid dysfunction can predict response to immunotherapy with interleukin-2 and interferon-2 alpha.

**DOI:** 10.1038/bjc.1991.426

**Published:** 1991-11

**Authors:** I. Reid, I. Sharpe, J. McDevitt, W. Maxwell, R. Emmons, W. A. Tanner, J. R. Monson

**Affiliations:** Department of Surgery, Meath Hospital, Dublin, Ireland.

## Abstract

Thyroid dysfunction is a well-recognised side-effect of treatment with interleukin-2 (IL2). We assessed the correlation between the development of abnormal thyroid function and tumour response in 13 patients receiving IL2 and interferon-2 alpha (IFN2 alpha) for advanced malignancy. Seven patients had normal thyroid function during treatment, and all of these patients have since died of progressive disease. Of six patients who did develop thyroid dysfunction during treatment, one patient has died of progressive disease. However, statistically we were unable to confirm a definite correlation between the development of thyroid dysfunction and survival in this small group of patients.


Br. J. Cancer (1991), 64, 915–918 © Macmillan Press Ltd., 1991

### Thyroid dysfunction can predict response to immunotherapy with interleukin-2 and interferon-2α

I. Reid, I. Sharpe, J. McDevitt, W. Maxwell, R. Emmons, W.A. Tanner & J.R.T. Monson

*Department of Surgery, Meath Hospital, Dublin 4, Ireland and F. Hoffman La-Roche, Basle, Switzerland*

**Summary** Thyroid dysfunction is a well-recognised side-effect of treatment with interleukin-2 (IL2). We assessed the correlation between the development of abnormal thyroid function and tumour response in 13 patients receiving IL2 and interferon-2α (IFN2α) for advanced malignancy.

Seven patients had normal thyroid function during treatment, and all of these patients have since died of progressive disease. Of six patients who did develop thyroid dysfunction during treatment, one patient has died of progressive disease. However, statistically we were unable to confirm a definite correlation between the development of thyroid dysfunction and survival in this small group of patients.

Over the past 14 years Interleukin-2 (IL-2) has been extensively investigated as an anti-tumour agent. Its main role at present is in the treatment of metastatic renal cell carcinoma and malignant melanoma, where IL-2 has been used alone and also in combination with other agents such as interferon, lymphokine-activated killer (LAK) cells or tumour-infiltrating lymphocytes (TIL). Documented response rates in renal cell carcinoma and malignant melanoma in patients treated with IL-2, or with combinations of IL-2 and one of the above agents, range from 20–40% (Rosenberg *et al.*, 1987; Rosenberg *et al.*, 1989). Treatment with IL-2 is associated with a well-documented range of side-effects (Lotze *et al.*, 1986*b*; Parkinson, 1988; Lotze *et al.*, 1986*a*). Abnormalities of thyroid function in association with IL-2 treatment were first noted by Atkins *et al.* in 1988. The reason for this association is not clear, but of particular interest is the suggestion that the development of thyroid dysfunction may be a marker of response to IL-2 treatment.

This report assesses the correlation between thyroid function and tumour response in a group of patients treated with IL-2 and Interferon-2α (IFN-2α) for disseminated malignant disease.

### Methods

Patients were treated as part of an open non-randomised study using a combination of IL-2 and IFN-2α. Patients eligible for this study had histologically confirmed, progressive, metastatic or recurrent malignant melanoma or renal cell carcinoma unsuitable for further surgery. All patients with renal cell carcinoma had previously undergone nephrectomy.

Each patient gave written informed consent to entry into the study and the study protocol was approved by the Institutional Review Board and Ethics Committee of the hospital.

### *Treatment protocol*

The treatment protocol was based on a 14 day treatment cycle (IL-2 and IFN-2α were provided by Roche Ireland Ltd, Bray, Co Wicklow). IL-2 at 3 MU $m^{-2}$ $day^{-1}$ was given by continuous intravenous infusion over days 1–4 (96 h). IFN-2α 6 MU $m^{-2}$ $day^{-1}$ was given as a subcutaneous bolus injection on days 1 and 4. There was then a 10 day rest period, and the cycle began again on the 15th day. IL-2 was administered via central venous access line and regulated by a continuous infusion pump.

Tumour measurement was performed after the second and fourth treatment cycles. After four cycles patients with progressive disease were withdrawn from the study. Patients with stable disease or better continued for a further nine cycles to a total of 13 cycles. Tumour assessment was then performed at monthly intervals and patients with documented progressive disease were withdrawn from the study at the time of diagnosis. After 13 cycles treatment ceased in patients with progressive disease or stable disease. Patients with complete or partial response continued treatment for a further 13 cycles up to a total of 26 cycles.

### *Thyroid function*

Serum thyroxin (T4) and thyrotropin (TSH) were measured by radioimmunoassay. Serum levels of thyroid antibodies (anti-thyroglobulin and anti-thyroid microsomal antibody) were measured by passive haemagglutination with commercial kits (Wellcome Ltd, Airton Road, Tallaght, Dublin 24). Anti-microsomal antibody titre above 1:100 and anti-thyroglobulin antibody titre above 1:10 were considered elevated. Thyroid function tests were performed on all patients prior to entry into the study and on days 1 and 4 of each treatment cycle. Thyroid antibody levels were measured prior to entry into the study, and subsequently on alternate treatment cycles (i.e. at monthly intervals) while on treatment.

### *Statistical analysis*

Differences in survival between groups were analysed using a log-rank test. Further statistical analysis of these data was complicated by the fact that patients failing to respond to treatment were withdrawn after two treatment cycles, while those patients responded to treatment continued on study. In order to determine whether abnormal thyroid function was a predictor of survival, while allowing for the longer treatment period in responding patients, we used a time-dependent Cox model to analyse these data.

### Results

Seventeen patients have been treated according to this protocol. One patient had a history of previous thyroidectomy and was found to be hypothyroid prior to commencing treatment, and was excluded from further analysis. Three patients received two treatment cycles or fewer. All three were in progressive disease at the time of withdrawal from the study, and all have since died of progressive disease. Thyroid function tests on these three patients were normal while on treatment. However, as thyroid dysfunction did not

Correspondence: J.R.T. Monson, Academic Surgical Unit, Queen Elizabeth the Queen Mother Wing, St Mary's Hospital, London W2 1NY, UK.
Received 18 January 1991; and in revised form 9 July 1991.

develop in most patients until the third treatment cycle or later, these three patients were also excluded from further analysis. Thirteen patients were therefore evaluated for this study. The clinical characteristics are summarised in Table I.

The median age of the group was 59 years (range 32–70 years). All patients had normal T4 and TSH levels at entry into the study, and no patient had anti-thyroid antibodies detected. None of the patients had a previous history of thyroid disease or thyroid surgery.

The 13 patients received a total of 104 treatment cycles (median seven cycles; range 3–15 cycles). Six patients (46%) developed biochemical thyroid dysfunction during treatment (defined as T4 or TSH levels outside the normal range of our laboratory on two consecutive treatment cycles). The remaining seven patients (54%) had no abnormalities of thyroid function detected. None of the 13 patients had anti-thyroid antibodies detected at any time during their course of treatment.

There was no correlation between age, sex, type of malignancy or Karnofsky Index and the development of thyroid dysfunction. The pattern of thyroid dysfunction which developed during treatment with IL-2 and IFN2α was similar in all six patients (Figure 1). Four patients developed elevated T4 with sub-normal TSH levels during the second to fifth treatment cycles. Three of these patients then became hypothyroid between the sixth and ninth cycles, and were commenced on replacement treatment with thyroxine. The fourth patient had an initial transient rise in T4, but subsequently maintained a normal T4 level with markedly elevated TSH (8 to 28: normal range 0.5–5). A fifth patient had a initial fall in TSH on the fourth and fifth cycles without a rise in T4. He subsequently became hypothyroid on the ninth cycle and was commenced on thyroxine. The last patient deviated slightly from this pattern in that his thyroid function remained normal up to cycle eight. He then developed raised T4 with low TSH levels. This patient is still on treatment.

Altogether, four patients required replacement therapy with thyroxine. Two patients had abnormalities of thyroid function, but did not become hypothyroid.

*Tumour response*

Five patients had progressive disease with no response to treatment and were therefore withdrawn from the study after two months (four treatment cycles). All of these patients have since died of progressive disease.

One patient had stable disease and continued on treatment for a total for 5 months (13 treatment cycles). She then developed refractory hypotension associated with IL-2 infusion, and was withdrawn from further treatment. A second patient was assessed as having stable disease after four treatment cycles, but was withdrawn from the treatment protocol at her own request. Both these patients have since died of progressive disease.

Two patients with stable disease at 6 months (12 cycles) were withdrawn from treatment at that point. One of these patients is now disease-free after surgical resection of residual disease. The second patient has since developed progressive disease. One patient had an initial partial response, but relapsed while on treatment and was withdrawn at 6 months (12 cycles).

Three patients achieved a partial response after 6 months on treatment. One of these has since developed progressive disease, but the two partial responses remain. Of the 13 patients, four partial responses were observed (30.8%), with four patients (30.8%) achieving stable disease while on treatment. Five patients (38.5%) had progressive disease with no response to treatment. There were no complete responses.

*Correlation between thyroid function and tumour response (Table II)*

Of the six patients with abnormalities of thyroid function, all had either stable disease or a partial response.

Seven patients had normal thyroid function throughout their treatment, two (29%) experiencing stable disease while on treatment.

*Survival and thyroid function*

Of six patients who developed thyroid dysfunction while on treatment, five patients (83%) were alive at the time of writing. The median survival in this group to date is 13.5 months (range 8–20 months) (Figure 2).

All those who maintained normal thyroid indices have died between 1.5 and 7 months after starting therapy. The median survival of this group is 3 months (range 1.5–7 months).

**Figure 1** T4/TSH levels during IL2 and IFN-2α therapy.

**Table I** Patient characteristics

| Patient | Age | Sex | KI | Primary disease | Site of metastases |
|---|---|---|---|---|---|
| 1 | 32 | F | 80 | Melanoma | Multiple |
| 2 | 44 | M | 90 | Renal | Pulmonary |
| 3 | 55 | F | 90 | Melanoma | Cutaneous |
| 4 | 65 | M | 90 | Renal | Lymphadenopathy |
| 5 | 58 | M | 90 | Renal | Pulmonary |
| 6 | 45 | M | 90 | Renal | Multiple |
| 7 | 48 | F | 80 | Renal | Multiple |
| 8 | 70 | F | 80 | Renal | Multiple |
| 9 | 64 | F | 80 | Renal | Multiple |
| 10 | 62 | F | 70 | Melanoma | Intra-abdominal |
| 11 | 59 | F | 90 | Melanoma | Multiple |
| 12 | 66 | M | 70 | Melanoma | Multiple |
| 13 | 63 | M | 80 | Renal | Multiple |

KI = Karnofsky Index at entry. Multiple = Recurrent disease affecting more than one body system.

**Table II** Thyroid dysfunction and outcome

| Patient | Response | Current status | Thyroid function | Cycle* | Duration of treatment |
|---|---|---|---|---|---|
| 1 | SD | ALIVE | ABN | 4 | 14 |
| 2 | PR/PD | DEAD | ABN | 3 | 6 |
| 3 | PR | ALIVE | ABN | 2 | 13 |
| 4 | SD/PD | ALIVE | ABN | 4 | 13 |
| 5 | PR | ALIVE | ABN | 3 | 9 |
| 6 | PR | ALIVE | ABN | 8 | 13 |
| 7 | SD | DEAD | N | / | 8 |
| 8 | SD | DEAD | N | / | 4 |
| 9 | PD | DEAD | N | / | 2 |
| 10 | PD | DEAD | N | / | 4 |
| 11 | PD | DEAD | N | / | 4 |
| 12 | PD | DEAD | N | / | 3 |
| 13 | PD | DEAD | N | / | 4 |

PR = partial response; SD = stable disease; PD = progressive disease; PR/PD = initial partial response followed by progressive disease; ABN = abnormal; N = normal. *Refers to cycle at which thyroid dysfunction was first noted. Duration of treatment in cycles.

**Figure 2** Overall survival.

Using a log-rank test, the difference in survival between these two groups is significant with $P = 0.049$. However, using a time dependent Cox model to determine whether the development of thyroid dysfunction was a positive predictor of survival, the difference between the groups was not significant ($P = 0.7075$), i.e. there was no significant correlation between survival and the development of thyroid dysfunction.

### Discussion

The development of abnormal thyroid function has been well documented in association with treatment with alpha-interferon alone, IL-2 alone, IL-2 plus LAK cells, and IL-2 plus alpha-interferon (Atkins *et al.*, 1988; Pichert *et al.*, 1990). The mechanism responsible for this phenomenon has not been fully elucidated, but it is probably an auto-immune phenomenon due to the induction of HLA class II antigens on thyroid epithelial tissue (Atkins *et al.*, 1988; Pichert *et al.*, 1990). The aim of this study was to examine the possible correlation between the development of abnormal thyroid function and tumour response in a group of patients receiving IL-2 and IFN-2α for advanced malignancy.

In this prospective study of 17 patients serum T4 and TSH levels were measured every 2 weeks while on treatment. It is unlikely that transient abnormalities of thyroid function were missed in any patient. All patients had normal thyroid function prior to entry into the study.

Six of 13 patients developed some abnormality of thyroid function during treatment, and it was particularly striking that the pattern of thyroid abnormality which developed was similar in all these patients. This pattern has been previously noted (Pichert *et al.*, 1990) in a study using the same protocol of therapy and correlates well with an auto-immune pattern. It is therefore of interest that none of these patients had anti-thyroid antibodies detected. The other seven patients had no abnormalities of thyroid function detected.

When survival is related to thyroid function, there is an obvious correlation between the development of abnormal thyroid function and longer survival. The median survival of those patients developing thyroid dysfunction was more than four times that of patients with normal thyroid status. However, the statistical analysis of this data is complicated by the longer time on treatment of responding patients. The point at issue is whether the development of abnormal thyroid function which occurred in these patients was due simply to treatment with IL2 and IFN-2α, or whether it truly predicts a tumour response to treatment. In five out of six patients abnormal thyroid function developed early in the course of treatment, between the second and fourth treatment cycles, and did not require long-term treatment with IL2 and IFN-2α (Table II). This would support the contention that abnormal thyroid function is a predictor of disease response, rather than a consequence of long-term treatment.

To allow for the fact that responding patients received a longer course of treatment than non-responder, we used a time-dependent Cox model for statistical analysis. Using this model in this small group of patients we were unable to confirm statistically that abnormal thyroid function was a predictor of disease response.

Other studies have reported abnormal thyroid function in patients treated with IFN-2α alone. Burman *et al.* (1986) noted thyroid dysfunction in seven out of 39 patients receiving human leukocyte-derived alpha-interferon (huLe-IFN) for carcinoid tumours. Fentiman *et al.* (1988) reported the development of hypothyroidism in three out of ten patients treated with huLe-IFN for locoregional recurrence of breast carcinoma. However, thyroid dysfunction has not been associated with recombinant IFN-2α used alone, and it has been suggested that thyroid dysfunction with huLe-IFN is due to the presence of small amounts of gamma-interferon in the huLe-IFN preparation (Burman *et al.*, 1986).

It is not clear why immunotherapy with IL-2 and IFN-2α should induce auto-immune thyroiditis but not other forms of auto-immune disease. A crucial step in the initiation of an immune response is the recognition by antigen-specific T-cells of MHC class II molecules on the surface of antigen presenting cells. Normal thyroid cells do not express MHC class II antigens; in contrast, thyrocytes from patients with auto-immune thyroid disease have been shown to do so (Hanafusa *et al.*, 1983). HLA class II expression can be induced on cultured thyroid cells by the addition of IFN-gamma, but not by adding IFN-alpha or IL-2 without IFN-gamma (Todd *et al.*, 1985). One action of IL-2 however is to induce IFN-gamma production by T-lymphocytes, and possibly also by NK cells (Kasahara *et al.*, 1983). Development of auto-immune thyroiditis after IL-2 therapy may therefore be due to IFN-gamma production from activated T-lymphocytes, which results in MHC class II antigen expression on thyroid tissue.

Cohen *et al.* (1987) have reported a correlation between HLA-DR expression on tumour cells and response to therapy with IL-2 plus LAK. It therefore appears that response to therapy with IL-2 may correlate with the expression of HLA class II antigens on both tumour cells and thyroid tissue. However, whereas Cohen *et al.* reported that four out of five responding tumours were HLA-DR positive before therapy, none of our patient had evidence of thyroiditis until after commencing IL-2 therapy.

Pichert *et al.* (1990) performed fine needle aspiration cytology (FNAC) in three patients who developed thyroid dysfunction while undergoing treatment with IL-2 and IFN. All three patients were reported as having evidence of chronic thyroiditis, and all three had strong expression of HLA-DR antigen on thyroid tissue. We did not obtain FNAC on our series of patients, but in future it would be of interest to perform thyroid FNAC prior to starting treatment and again after the appearance of thyroid dysfunction. It may be that the presence of HLA-DR antigen on thyroid tissue will predict response to therapy.

It is clear from this study that patients undergoing treatment with IL-2 and IFN are at a high risk of developing thyroid dysfunction. Such patients should have regular assessment of thyroid function, and may require replacement therapy with thyroxine. However, as not all patients become hypothyroid, and as some patients may pass through an initial transient episode of hyperthyroidism before becoming hypothyroid, prophylactic therapy with thyroxine for all patients receiving IL-2 and IFN is not recommended.

Thyroid dysfunction is a common side-effect of therapy with IL2 plus IFN-alpha. In this small group, patients who developed abnormal thyroid function while on treatment with IL2 and IFN-2α had longer survival and were more likely to respond to treatment than patients without thyroid dysfunction, although in such a small number of patients we have been unable to definitively correlate abnormal thyroid function with tumour response.

The development of thyroid dysfunction may be associated with tumour response to treatment. The mechanisms of this response, and the usefulness of HLA-DR expression on tumour or thyroid tissue as a predictor of response to therapy requires further evaluation.

### References

ATKINS, M.B., MIER, J.W., PARKINSON, D.R., GOULD, J.A., BERKMAN, E.M. & KAPLAN, M.M. (1988). Hypothyroidism after treatment with interleukin-2 and lymphokine-activated killer cells. *N. Eng. J. Med.*, **318**, 1557.

BURMAN, P., TOTTERMAN, T.H., OBERG, K. & KARLSSON, F.A. (1986). Thyroid auto-immunity in patients on long-term therapy with leukocyte-derived interferon. *J. Clin. Endocrinol. Metab.*, **63**, 1086.

COHEN, P.J., LOTZE, M.T., ROBERTS, J.R., ROSENBERG, S.A. & JAFFE, E.S. (1987). The immunopathology of sequential tumour biopsies in patients treated with interleukin-2. Correlation of response with T-cell infiltration and HLA-DR expression. *Am. J. Pathol.*, **129**, 208.

FENTIMAN, I.S., BALKWILL, F.R., THOMAS, B.S., RUSSELL, M.J., TODD, I. & BOTTAZZO, G.E. (1988). An auto-immune aetiology for hypothyroidism following interferon therapy for breast cancer. *Eur. J. Cancer Clin. Oncol.*, **24**, 1299.

HANAFUSA, T., CHIOVATO, L., DONIACH, D., PUJOL-BORRELL, R., RUSSELL, R.C.G. & BOTTAZZO, G.F. (1988). Aberrant expression of HLA-DR antigen on thyrocytes in Graves' disease: relevance for autoimmunity. *Lancet*, **ii**, 1111.

KASAHARA, T., HOOKS, J.J., DOUGHERTY, S.F. & OPPENHEIM, J.J. (1983). Interleukin-2 mediated immune interferon-gamma production by human T cells and T cell subsets. *J. Immunol.*, **130**, 1784.

LOTZE, M.T., MATORY, Y.L. & RAYNER, A.A. (1986*a*). Clinical effects and toxicity of interleukin-2 in patients with cancer. *Cancer*, **58**, 2764.

LOTZE, M.T., CHANG, A.E., SCIPP, C.A., SIMPSON, C., VETTO, J.T. & ROSENBERG, S.A. (1986*b*). High dose recombinant interleukin-2 in the treatment of patients with disseminated cancer: responses, treatment-related morbidity and histologic findings. *JAMA*, **256**, 3117.

PARKINSON, D.R. (1988). Interleukin-2 in cancer therapy. *Seminars in Oncol.*, **15**, Suppl 6, 10.

PICHERT, G., JOST, L.M., ZOBELI, L., ODERMATT, B., PEDIO, G. & STAHEL, R.A. (1990). Thyroiditis after treatment with interleukin-2 and interferon alpha-2a. *Br. J. Cancer*, **62**, 100.

ROSENBERG, S.A., LOTZE, M.T., MUUL, L.M. & 8 others (1987). A progress report on the treatment of 157 patients with advanced cancer using lymphokine-activated killer cells and interleukin-2 or high-dose interleukin-2 alone. *N. Engl. J. Med.*, **316**, 889.

ROSENBERG, S.A., LOTZE, M.T., YANG, J.C. & LINEHAN, W.M. (1989). Combination therapy with interleukin-2 and alpha-interferon for the treatment of patients with advanced cancer. *J. Clin. Oncol.*, **7**, 1863.

TODD, I., PUJOL-BORRELL, R., HAMMON, L.J., BOTTAZZO, G.F. & FELDMAN, M. (1985). Interferon-gamma induces HLA-DR expression by thyroid epithelium. *Clin. Exp. Immunol.*, **61**, 265.